# Changes in the Anterior Lamina Cribrosa Morphology with Glaucoma Severity

**DOI:** 10.1038/s41598-019-42649-1

**Published:** 2019-04-29

**Authors:** Nicholas Y. Q. Tan, Yih-Chung Tham, Sri Gowtham Thakku, Xiaofei Wang, Mani Baskaran, Marcus C. L. Tan, Jean-Martial Mari, Nicholas G. Strouthidis, Tin Aung, Michaël J. A. Girard, Ching-Yu Cheng

**Affiliations:** 1 Singapore Eye Research Institute, Singapore National Eye Centre, Singapore, Singapore; 2 Department of Biomedical Engineering, National University of Singapore, Singapore, Singapore; 3 Ophthalmology and Visual Sciences Academic Clinical Program, Duke-NUS Medical School, Singapore, Singapore; 4 Department of Ophthalmology, Yong Loo Lin School of Medicine, National University of Singapore, Singapore, Singapore; 5 Vision Performance Centre, Singapore Armed Forces, Singapore, Singapore; 6 GePaSud, Université de la Polynésie Française, Tahiti, French Polynesia; 7 NIHR Biomedical Research Centre, Moorfields Eye Hospital NHS Foundation Trust and UCL Institute of Ophthalmology, London, UK

**Keywords:** Biomarkers, Translational research

## Abstract

This study was designed to evaluate if primary open angle glaucoma (POAG) and its severity are associated with the shape of the lamina cribrosa (LC) as measured by a global shape index (LC-GSI), or other indices of LC curvature or depth. Optical coherence tomography (OCT) scans of the optic nerve head (OHN) were obtained from subjects with POAG (n = 99) and non-glaucomatous controls (n = 76). ONH structures were delineated, the anterior LC morphology reconstructed in 3D, and the LC-GSI calculated (more negative values denote greater posterior concavity). Anterior LC depth and 2D-curvature were also measured. Severity of glaucoma was defined by the extent of visual field loss, based on the Hodapp-Parrish-Anderson grading. Linear regression analyses compared LC characteristics between controls, mild-moderate, and advanced POAG groups. After adjusting for age, gender, ethnicity, intraocular pressure, axial length and corneal curvature, the LC-GSI was most negative in the advanced POAG group (mean [standard error] = −0.34 [0.05]), followed by the mild-moderate POAG group (−0.31 [0.02]) and then controls (−0.23 [0.02], *P*_Trend_ = 0.01). There was also a significant trend of increasing LC depth and greater LC horizontal curvature with increasing severity of glaucoma (*P*_Trend_ = 0.04 and 0.02, respectively). Therefore, with more severe glaucoma, the LC-GSI was increasingly more negative, and the anterior LC depth and curvature greater. These observations collectively correspond to greater cupping of the ONH at the level of the LC. As the LC-GSI describes the 3D anterior LC morphology, its potential usage may be complementary to existing ONH parameters measured on OCT.

## Introduction

The lamina cribrosa (LC) is a reticulated, sieve-like structure that fills the posterior scleral foramen, which unmyelinated retinal ganglion cell (RGC) axons pass through before converging as the optic nerve^1,2^. As the LC is subject to mechanical strains as it straddles two differentially pressured compartments (intraocular pressure [IOP] anteriorly, and retrobulbar cerebrospinal fluid pressure posteriorly)^3^, the LC is thought to be the main site of pressure-related RGC axonal insult in glaucoma^4,5^. Defining the structural characteristics of the LC is therefore important for a few interrelated reasons. First, when the LC is subject to pressure-related mechanical strain in glaucoma, the LC may deform^6,7^. Thus, being able to detect structural changes to the LC may enable us appraise the biomechanical responses of the LC^8,9^. Second, structural features of the LC may also indicate the susceptibility of the optic nerve head (ONH) to glaucomatous damage. For instance, LC thickness and posterior displacement of the LC were both associated with the rate of progressive retinal nerve fibre layer (RNFL) thinning^10^. Third, structural characteristics of the LC may also be indicative of the severity of glaucoma^11,12^. It is well known that the extent of visual field loss has good correspondence to the amount of cupping of the optic disc—this is in part due to tissue remodelling of the LC^13,14^.

As the LC is not readily visible on ophthalmoscopy due to the presence of overlying prelaminar neuroretinal tissue, *in vivo* structural examination of the LC has not been feasible until recently with advances in optical coherence tomography (OCT) imaging. Existing LC parameters that are commonly reported using OCT include LC depth^11,15^ and LC curvature^16,17^. However, describing the LC in only 1-dimension (D) (depth) or 2D (curvature) is poorly reflective of the LC as a complex 3D biomechanical structure. To this end, our group has previously described a new morphologic measure of the LC termed the “LC global shape index” (LC-GSI), which characterizes the overall geometrical shape of the anterior LC surface from a 3D ONH reconstruction^18^. Although the LC-GSI was developed from a set of healthy non-glaucomatous eyes, significant associations were demonstrated between the LC-GSI and traditional biomarkers of glaucoma such as the vertical cup-disc ratio (VCDR) and the minimum rim width^18,19^. Therefore, in the present study, we have applied this previously reported methodology to characterize the shape and morphology of the anterior LC in an independent set of control and glaucomatous eyes (of varying disease severity), to assess the association of LC-GSI with glaucoma. In addition, existing LC parameters such as its depth and curvature were also similarly measured and reported.

## Methods

### Study population

This study involved a total of 142 subjects with primary open angle glaucoma (POAG) and 88 controls without glaucoma, and was conducted at the Singapore Eye Research Institute (SERI) between June 2013 and February 2016. Individuals diagnosed with POAG were recruited from glaucoma clinics at the Singapore National Eye Centre between April 2015 and February 2016. Non-glaucomatous controls aged 50 years and older were recruited between August to September 2013 from Outram Polyclinic, a government-run primary care clinic in the central region of Singapore, where they attended regular medical appointments for non-ophthalmic issues. Written informed consent was obtained from each participant. The study adhered to the tenets of the declaration of Helsinki, and ethics committee approval was provided by the SingHealth Centralised Institutional Review Board.

### Ophthalmic assessment

All subjects underwent a standardized ophthalmic examination. Best-corrected visual acuity was measured on a logMAR chart. Refractive error and corneal curvature were measured using an autorefractor (Canon RK 5 Auto Ref-Keratometer; Canon Inc., Ltd., Tochigiken, Japan). Central corneal thickness was measured with an ultrasound pachymeter (Advent; Mentor O & O Inc., Norwell, MA). Axial length was measured using non-contact partial coherence interferometry (IOL Master V3.01, Carl Zeiss Meditec AG, Jena, Germany). Intraocular pressure (IOP) was measured using the Corvis ST Tonometer (Oculus, Wetzlar, Germany). Subjects were also examined by an ophthalmologist under the slit lamp for anterior segment examination, gonioscopy, and optic disc examination through a dilated pupil with the 78-diopter (D) lens at x16 magnification with a measuring graticule. Optical imaging of the lamina cribrosa was performed as described below. Subjects with glaucoma underwent visual field (VF) assessment on standard automated perimetry (Swedish Interactive Threshold Algorithm Standard algorithm with a 24-2 test pattern, Humphrey Visual Field Analyser II; Carl Zeiss Meditec, Dublin, California, USA).

### Definitions of primary open angle glaucoma and controls

POAG was defined by the following criteria: the presence of glaucomatous optic neuropathy (defined as a VCDR > 0.7, VCDR asymmetry > 0.2, and/or focal notching) with a compatible VF defect, non-occludable angles on gonioscopy, and absence of secondary causes of glaucoma. All eyes with POAG included did not have prior surgical or laser treatment for glaucoma, but were on topical IOP-lowering medications. Severity of POAG was further defined by the extent of glaucomatous VF loss, based on the Hodapp-Parrish-Anderson grading scale^20^. Namely, mild-moderate POAG was defined where mean deviation (MD) value was −12.00 dB or better, and advanced POAG was defined where the MD was −12.01 dB or worse. Diagnoses were made from reliable VF results (where the fixation losses were less than 20%, and false-positive, false-negative rates were less than 33%).

All control subjects had bilateral eyes with best-corrected visual acuity ≤0.3, IOP ≤ 21 mmHg, VCDR ≤ 0.6 (without any fellow eye asymmetry of >0.2), absence of any signs consistent with secondary glaucomas, absence of significant cataract (defined as presence of cortical or posterior subcapsular cataract graded ≥ 2, or nuclear cataract graded ≥4 on the Lens Opacities Classification System III), and absence of any signs indicative of corneal, macular, vitreoretinal or optic nerve pathology.

### Optical coherence tomography imaging

Spectral-domain OCT raster scans were acquired for both ONHs of each subject. The eye tracking and enhanced depth imaging^21^ modalities of the Spectralis software were used during image acquisition. Each set of images comprised 97 serial horizontal B-scans (each composed of 384 A-scans) covering a rectangular region of 15 × 10° centered on the ONH. Distance between consecutive scans varied slightly across different eyes, with a mean of about 30 μm. The lateral resolution of each B-scan was roughly 12 μm horizontally and 3.9 μm axially. To reduce speckle noise, each B-scan was averaged 20 times during image acquisition.

### Image delineation and 3D reconstruction

Where more than one eye was eligible per subject, one of the two eyes were chosen using a random number generator on MATLAB (Mathworks Inc., Natick, MA) and delineated for further analysis. The delineation and 3D reconstruction process was based on our protocol described previously (Supplemental Fig. S1)^18^. Briefly, LC visibility in the raw OCT B-scans was first enhanced using adaptive compensation^22,23^. Enhanced images were then loaded on to a custom-written application on MATLAB and the following structures were delineated: (1) the anterior LC surface; (2) Bruch’s membrane opening (BMO); and (3) the inner limiting membrane. Using these delineations, we used a smoothing spline function to generate a best-fitting surface. This enabled a reconstruction of the ONH structures in 3D, and our customized MATLAB algorithms automatically derived the LC-GSI and other LC parameters according to established protocols and as described below^18^.

### Measurement of lamina cribrosa morphological parameters

After delineation and reconstruction, LC parameters were calculated for the portion of the LC underneath a circular region of radius 750 μm and centered on the BMO center (roughly 80% of average BMO area). This maintained consistency and ensured that measurements were made for similar portions of the LC across eyes. Since LC visibility varied across different eyes, particularly in the peripheral region, eyes with a poorly visible LC (where the LC covered less than 70% of the BMO area from *en face* visualization) were excluded from the analysis. Additionally, we excluded eyes with extensive peripapillary atrophy as this would result in difficulty in discerning the BMO boundaries. We defined and calculated in MATLAB the following LC morphological parameters^18^.

**Figure 1:**
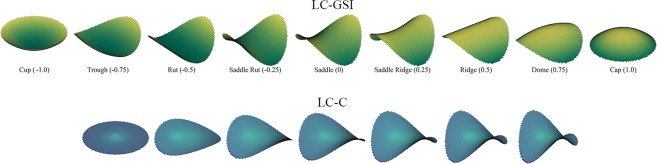
Illustration of the lamina cribrosa global shape index (LC-GSI) and the lamina cribrosa curvedness (LC-C). (**A**) The local surface shape of the anterior lamina cribrosa is divided into nine categories, with the LC-GSI in parentheses: cup, trough, rut, saddle rut, saddle, saddle ridge, ridge, dome and cap. The insert figures are shapes of identical curvedness. (**B**) A symmetrical saddle of equal shape index is depicted at increasing LC-C values: lowest on the left (zero), and highest on the right.

The LC-GSI is a number from the range of −1 (corresponding to a spherical cup) to + 1 (corresponding to a spherical cap) that describes the local surface shape of the anterior LC (Fig. 1). It is independent of the size of the LC, and is derived from the maximum (K_1_) and minimum (K_2_) principal arc curvatures of the LC, according to the equation^18,24^ below:

1 $${\rm{LC}}-{\rm{GSI}}=\frac{2}{\pi }{\tan }^{-1}\frac{{\kappa }_{1}+{\kappa }_{2}}{{\kappa }_{1}-{\kappa }_{2}}\,$$

In contrast to the LC-GSI which specifies the type of shape the LC corresponds to, and is scale-independent, the LC-Curvedness (LC-C) is a positive number that indicates the “intensity” of the surface curvature, and is scale-dependent (Fig. 1). The LC-C is also derived from the principal arc curvatures K_1_ and K_2_ according to the equation^24^ below:

2 $${\rm{LC}}\,-\,{\rm{C}}=\sqrt{\frac{{\kappa }_{1}^{2}+{\kappa }_{2}^{2}}{2}}$$

Considered together, the LG-GSI and the LC-C specify the local second order 3D geometry of the LC. For example, a LC with a LC-GSI of 0 indicates that it is best described by a “saddle” shape, and an accompanying LC-C of 1.2 describes how intensely “saddle”-shaped it is (the rightmost example in the bottom panel of Fig. 1 in this instance).

Additionally, further measures of the curvature of the LC as assessed in a 2D plane were also reported: curvature in the nasal-temporal plane (N-T Curvature), and in a superior-inferior plane (S-I Curvature)^18^. To derive these parameters, we first intersected the anterior LC surface with horizontal (N-T) and vertical (S-I) radial cross-sections that passed through the center of the BMO ellipse, and were perpendicular to the BMO plane. A circular arc was then fitted to each LC curve. A positive or negative (N-T or S-I) arc curvature indicated the degree to which it was curved anteriorly or posteriorly, respectively.

**Figure 2:**
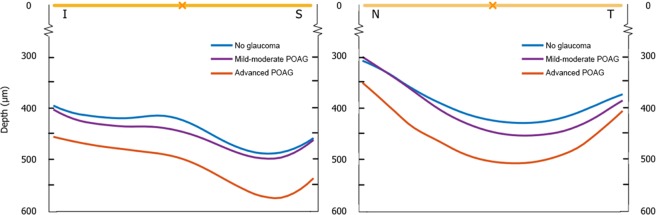
Anterior lamina cribrosa (LC) depth along the superior-inferior (S-I) and nasal-temporal (N-T) axes across the three groups: healthy controls, mild-moderate primary open angle glaucoma (POAG) and advanced POAG. (**A**) Along the S-I direction, the LC is more posterior (deeper) with increasing severity of glaucoma; the central ridge also becomes less prominent, with the general shape changing from a “W” to a flatter “U”. (**B**) Along the N-T direction, the LC is overall “U”-shaped; the LC is also more posterior with increasing severity of glaucoma.

Finally, the depth of the anterior LC was defined as the distance from the BMO reference plane to anterior LC surface, reconstructed from each delineated LC point. The LCD was calculated from the mean of these measurements^18^. The anterior depth of the LC was also plotted along the vertical and horizontal axes, as illustrated in Fig. 2.

### Statistical analysis

Statistical analyses were performed using Stata Version 14.0 (StataCorp LP, College Station, TX). The Chi-square test (for gender), Fisher’s exact test (for ethnicity) and ANOVA (for continuous variables) were used to compare the demographics, clinical characteristics and LC parameters between the three groups: controls, mild-moderate POAG, and advanced POAG. Multivariable linear regression models were used to compare the difference in the LC parameters among the three groups, adjusting for age, gender, ethnicity, IOP, axial length and corneal curvature, and a test for trend (normal, mild-moderate glaucoma, followed by advanced glaucoma) was performed. *P* value for significance was set at <0.05.

## Results

Of the 230 subjects, after excluding 27 glaucomatous eyes and 12 control eyes due to poor visibility of the LC on OCT or extensive peripapillary atrophy and indistinct BMO boundaries, and 16 participants with missing key variables, a total of 175 eyes from 175 subjects were included in our analyses. Of these, 76 were controls, 82 were mild-moderate POAG (MD [standard deviation] = −5.34 [3.17] dB), and 17 were advanced POAG cases (−17.36 [4.00] dB). Table 1 shows the demographic and clinical characteristics of the three groups. Overall, POAG cases were more likely to be males, older, and with higher axial lengths and VCDR, compared to controls (all *P* ≤ 0.005). With POAG cases already being on medical therapy, IOP was not significantly different among the three groups (*P* = 0.35).

**Table 1:** Comparison of ocular and clinical parameters between control and glaucoma groups.

Parameters	No glaucoma (n = 76)	Mild-Moderate POAG (n = 82)	Advanced POAG (n = 17)	P*
Age, years	59.29 (6.25)	65.30 (8.88)	67.41 (4.35)	<0.001
Gender, female	44 (57.89)	34 (41.46)	3 (17.65)	0.005
Race				
Chinese	72 (94.74)	74 (90.24)	13 (76.47)	0.16
Malay	1 (1.32)	2 (2.44)	2 (11.76)	
Indian	2 (2.63)	4 (4.88)	2 (11.76)	
Others	1 (1.32)	2 (2.44)	0 (0.00)	
IOP, mmHg	14.71 (2.95)	14.83 (2.95)	16.00 (5.94)	0.35
Axial length, mm	23.82 (1.24)	24.83 (1.40)	24.15 (1.15)	<0.001
CCT, μm	543.33 (28.54)	534.80 (68.56)	523.50 (26.11)	0.30
CC, mm	7.64 (0.27)	7.70 (0.20)	7.73 (0.22)	0.21
VCDR	0.49 (0.13)	0.73 (0.10)	0.83 (0.08)	<0.001

Data are in mean (standard deviation) or *n* (%) as appropriate.

BCVA = best corrected visual acuity; CC = corneal curvature; CCT = central corneal thickness; IOP = intraocular pressure; POAG = primary open angle glaucoma; VCDR = vertical cup-to-disc ratio.

*Comparison between the three groups using the Chi-square test (for gender), Fisher’s exact test (for ethnicity) and ANOVA (for continuous variables).

Figure 2 depicts the morphology of the anterior LC surface in 2D by plotting the mean anterior LC depth along the S-I and N-T axes across the three groups. Along the S-I direction, the LC was overall “W”-shaped in normal controls, with a central hump, and depressions in the superior and inferior mid-periphery; however, with increasing severity of glaucoma, the LC was more posterior (deeper), and assumed a flatter “U”-shaped profile. Along the N-T direction, the LC was overall “U”-shaped across all 3 groups, and the LC was also more posterior with increasing severity of glaucoma.

The anterior LC morphology was further quantified by measuring the LC-GSI, LC-C, LCD, and N-T and S-I Curvatures (Table 2). The LC-GSI (standard error) was −0.23 (0.02) in controls, −0.31 (0.02) in mild-moderate POAG, and −0.34 (0.05) in advanced POAG. On multivariable analyses (adjusted for age, gender, ethnicity, IOP, axial length and corneal curvature), there was a significant trend of decreasing LC-GSI moving from controls, to mild-moderate POAG, to advanced POAG (*P*_Trend_ = 0.01). Furthermore, the adjusted LC-GSI was also smaller in both POAG groups when either POAG group was compared individually against controls (all *P* < 0.01). Similarly, the LCD and N-T Curvature also showed significant trends (deeper and more curved, respectively) moving from no glaucoma, to mild-moderate POAG, and to advanced POAG (all *P*_Trend_ < 0.05). There were significant differences in the LCD and N-T Curvature between the advanced POAG and control groups (all *P* < 0.05). Although the LC was increasingly less anteriorly curved along the S-I axis with increasing severity of glaucoma, this trend was not significant (*P*_Trend_ = 0.07). LC-C was also not associated with glaucoma.

**Table 2:** Comparison of lamina cribrosa parameters between control and glaucoma groups.

Parameters	No glaucoma (n = 76)	Mild-Moderate POAG (n = 82)	Advanced POAG (n = 17)	*P* for Trend^†^
LC-GSI	−0.23 (0.02)	−0.31* (0.02)	−0.34* (0.05)	0.01
LC-C	0.36 (0.01)	0.36 (0.01)	0.41 (0.03)	0.24
N-T Curvature, m^−1^	−424.6 (17.9)	−444.8 (17.1)	−537.7* (37.1)	0.02
S-I Curvature, m^−1^	190.4 (22.1)	131.3 (21.1)	117.5 (45.7)	0.07
LCD, µm	399.7 (10.2)	415.3 (9.7)	452.7* (21.0)	0.04

LC-C = lamina cribrosa curvedness; LC-GSI = lamina cribrosa global scale index; LCD = lamina cribrosa mean depth; N-T Curvature = nasal-temporal curvature; POAG = primary open angle glaucoma; S-I Curvature = superior-inferior curvature.

Data are presented as the mean values (standard error), adjusted for age, gender, race, intraocular pressure, axial length, corneal curvature.

*Denotes significant difference (on multivariable linear regression) when compared to the non-glaucoma group (*P* < 0.05).

^†^Comparison between the three groups using multivariable linear regression.

## Discussion

The LC-GSI was previously introduced as a new quantitative measure of anterior LC morphology independent from the BMO plane; it was developed from a set of healthy non-glaucomatous eyes, but yet showed associations with other structural biomarkers of glaucoma^18^. Hence, we have applied the LC-GSI alongside other LC-specific parameters in the comparison of healthy versus glaucomatous eyes. Here, we have found the LC-GSI to be significantly more negative (i.e. the LC more concave or cupped) in mild-moderate POAG (mean[standard error]: −0.31 [0.02]) and advanced POAG (−0.34 [0.05]) compared to controls (−0.23 [0.02]), with a significant trend moving across these 3 groups (from controls, to mild-moderate POAG to advanced POAG, *P*_Trend_ = 0.01). Notably, the LC-C was not significantly different between all groups. This suggests that the morphological changes to the LC in glaucoma manifests by primarily by a quality of shape, rather than intensity of shape—for instance, a glaucomatous change to the LC may involve changing from a “saddle” shape to a “trough” shape, rather than remaining as more intensely “saddle” shaped (Fig. 1). Viewed in a 2D vertical or horizontal profile, the LC was also increasingly posteriorly displaced and posteriorly curved in both axes, and the central hump^25^ in the vertical dimension was gradually diminished with increasing severity of glaucoma (Fig. 2)^16^. Existing LC-parameters such as the LCD and LC curvature also reflect these changes. We have found the anterior LC to be deeper (larger LCD), and the LC more posterior curved (N-T Curvature more negative) in glaucomatous compared to healthy eyes, consistent with prior reports^11,16,17^. These results underscore the notion that structural LC characteristics are an important aspect of ONH assessment in glaucoma.

Cupping of the ONH is a key feature of glaucoma that is of diagnostic importance, and is readily observable on ophthalmoscopy or on commonly used imaging techniques such as OCT. This cupping or excavation of the ONH may be attributable to two components: prelaminar and laminar^14,26^. It is proposed that the loss or thinning of the prelaminar neural tissues, which is a feature of both glaucomatous and non-glaucomatous optic neuropathies alike, results in a shallow increase in both the depth and width of the cup; however, this form of ONH cupping is non-specific, as it occurs in all forms of RGC axonal loss^27,28^. Deformation and connective tissue-based remodelling of the LC on the other hand, results in a deeper form of cupping that is thought to be more specific to glaucoma^13,14^. It is postulated that chronic pressure-related mechanical strain on the LC results in LC bowing back and assuming a more excavated appearance^13,14^.

The clinical assessment of glaucoma commonly involves imaging of the ONH using either spectral domain OCT or confocal scanning laser ophthalmoscopy, and the evaluation of various well-described optic disc morphological parameters such as neuroretinal rim volume, cup volume, and the cup-to-disc ratio. However, these parameters are either specific to prelaminar changes to the ONH (e.g. neuroretinal rim thinning), or do not discriminate between prelaminar or laminar changes (e.g. cup volume, cup-to-disc ratio). None describe the morphology of the LC itself. Although LC-parameters such as LCD and LC Curvature have previously been reported, describing the LC in only 1D or 2D does not capture the complexity of the LC as a 3D structure. In this regard, the LC-GSI is a valuable parameter as it offers a novel way of describing the 3D laminar changes to the ONH as a single metric; being a quantitative score, it is intuitive to understand and easy to apply in the evaluation of glaucoma.

In studies of non-human primates with experimental glaucoma, it was found that LC deformation could be observed before RNFL thinning occurred^29,30^. Furthermore, recent longitudinal clinical studies have also demonstrated that eyes with a greater baseline LC curvature^31^, or a greater baseline LD depth^10^, were associated with a faster rate of RNFL loss. Thus, detecting LC deformation with the LC-GSI may potentially serve as an early sign before the onset of the irreversible visual field defects and RNFL thinning. In a previous study, our group also showed that with acute IOP elevation, dynamic changes to the anterior LC surface were detectable using the LC-GSI parameter; however, the LCD did not show significant changes^7^. Additionally, LC curvature was also shown to be reduced after IOP-lowering with trabeculectomy^32^. Thus, it is possible that changes to the LC-GSI could also allude to changes in biomechanical (IOP-related) strains at the ONH^33^; however, this would require further study. In our present study, we have shown the LC-GSI to be significantly more negative (i.e. the LC more concave) with more severe glaucoma. However, additional prospective studies would be needed to determine whether the LC-GSI is a permanent indicator of the severity of glaucomatous change to the ONH, or whether it is an indicator of reversible strain at the ONH (that may be relieved with IOP-lowering therapy).

The findings of this study should be considered in the light of its limitations. First, our sample size of 76 healthy eyes and 99 glaucomatous eyes (of which only 17 were advanced) is relatively modest and does not allow us to establish a normative database. Second, majority of our subjects were of Chinese ethnicity—therefore, our findings may not be generalizable in other populations. Third, as part of our exclusion criteria for healthy eyes, we did not include any non-glaucomatous control eyes with VCDR of >0.6. Thus, a comparison of the LC-GSI of disc suspects against definite glaucoma could not be made. In future studies, it would be interesting to see if the LC-GSI may have discriminatory power to differentiate between the two. Fourth, although we found the LC-GSI to be significantly different in eyes with different severities of POAG compared to controls, the cross-sectional nature of our data limits inferences of causality—thus, we are unable to determine if the LC-GSI may progressively change with increasing severity of glaucoma^29^, or if the LC-GSI may be a risk factor for glaucomatous progression^10^, or both. Future studies that clarify these causal relationships may help establish the clinical utility of the LC-GSI parameter in the diagnosis or management of glaucoma. Fifth, we were able to account for only anterior LC morphology (of which LC-GSI is a measure), but not posterior LC morphology (or LC thickness) because of the poor visibility of the posterior LC surface with existing methods of OCT imaging^23^. However, limitations due to restricted visibility of the LC may potentially improve with newer imaging technologies with greater depth penetrance (e.g. swept-source OCT). Lastly, the LC-GSI is intended to be a global shape measure, and thus “smooths” out local variations in the LC (such as focal LC defects, which may be of pathological significance in glaucoma). The utility of an additional local shape measure that may be more sensitive to local variations in the LC would benefit from further study.

In conclusion, we have shown for the first time that the LC-GSI (a metric that describes the 3D anterior LC morphology) is more negative (i.e. the LC more cupped) in glaucomatous compared to non-glaucomatous eyes. Furthermore, there was a trend of decreasing LC-GSI moving from controls, to mild-moderate POAG, to advanced POAG. This therefore suggests that the LC-GSI may have a potential utility as an additional structural parameter of glaucoma. Our study provides the groundwork for future work in this area.

## Supplementary information

Supplemental Figure
