# Supplementary material for: Changes in the Anterior Lamina Cribrosa Morphology with Glaucoma Severity
Source: Sci Rep. 2019 Apr 29;9:6612. doi: 10.1038/s41598-019-42649-1 (PMC6488637; doi:10.1038/s41598-019-42649-1)
Supplement: Supplementary file 1 — Supplemental Figure [file 41598_2019_42649_MOESM1_ESM.pdf]

Supplementary Information for:
Changes in the Anterior Lamina Cribrosa Morphology with Glaucoma Severity

Nicholas Y.Q. Tan[a1]; Yih-Chung Tham[a1]; Sri Gowtham Thakku[1,2]; Xiaofei Wang[2]; Mani Baskaran[1,3]; Marcus C.L. Tan[4,5]; Jean-Martial Mari[6]; Nicholas G. Strouthidis[1,7]; Tin Aung[1,3,4]; Michaël J.A. Girard[1,2]; Ching-Yu Cheng*[1,3,4]

1. Singapore Eye Research Institute, Singapore National Eye Centre, Singapore
2. Department of Biomedical Engineering, National University of Singapore, Singapore
3. Ophthalmology and Visual Sciences Academic Clinical Program, Duke-NUS Medical School, Singapore
4. Department of Ophthalmology, Yong Loo Lin School of Medicine, National University of Singapore, Singapore
5. Vision Performance Centre, Singapore Armed Forces, Singapore
6. GePaSud, Université de la Polynésie Française, Tahiti, French Polynesia
7. NIHR Biomedical Research Centre, Moorfields Eye Hospital NHS Foundation Trust and UCL Institute of Ophthalmology, London, UK

[a]Both authors contributed equally as first authors.

***Correspondence to**: Prof Ching-Yu Cheng, Singapore Eye Research Institute, The Academia, 20 College Road, Discovery Tower, Level 6, Singapore 169856. Tel: +65 65767277, Fax: +65 6225 2568. Email: chingyu.cheng@duke-nus.edu.sg.

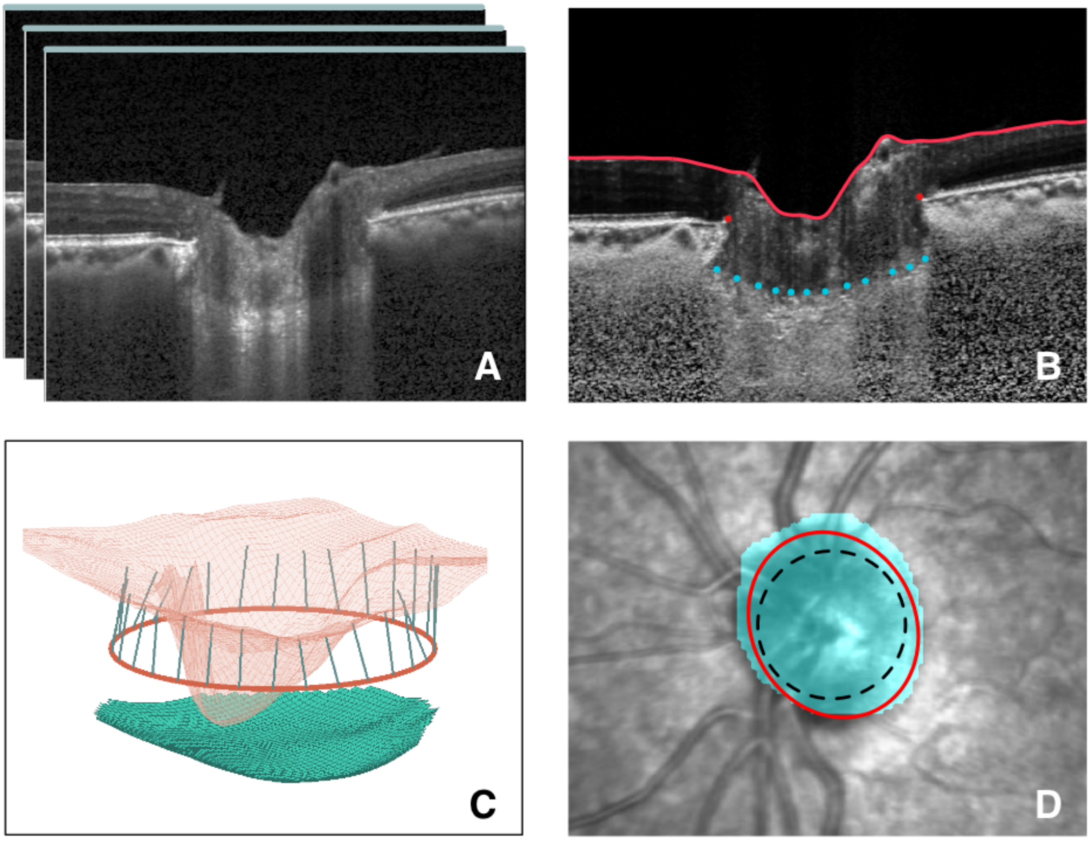

Supplemental Figure. Reconstruction of the anterior lamina cribrosa (LC) and optic nerve head (ONH) region based on optical coherence tomography B-scans. (**A**) A series of horizontal B-scans were centered on the ONH. (**B**) LC visibility was enhanced using adaptive compensation. The internal limiting membrane is denoted by the red line, the Bruch's membrane opening (BMO) by the two red points, and the anterior LC by the blue dots. (**C**) 3D reconstruction of the ONH region based on the delineations. (**D**) *En face* view of the BMO (red) and LC (blue). The dotted black line denotes a circle of radius 750 µm centered at the BMO center. LC parameters were computed for the portion of the LC underneath this circle.
